# Computational drug repositioning of atorvastatin for ulcerative colitis

**DOI:** 10.1093/jamia/ocab165

**Published:** 2021-09-16

**Authors:** Lawrence Bai, Madeleine K D Scott, Ethan Steinberg, Laurynas Kalesinskas, Aida Habtezion, Nigam H Shah, Purvesh Khatri

**Affiliations:** 1 Immunology Program, Stanford University School of Medicine, Stanford, California, USA; 2 Institute for Immunity, Transplantation and Infection, Stanford University School of Medicine, Stanford, California, USA; 3 Center for Biomedical Informatics Research, Department of Medicine, Stanford University, Stanford, California, USA; 4 Biophysics Program, Stanford University School of Medicine, Stanford, California, USA; 5 Computer Science Program, Department of Computer Science, Stanford University, Stanford, California, USA; 6 Biomedical Informatics Training Program, Stanford University School of Medicine, Stanford, California, USA; 7 Division of Gastroenterology and Hepatology, Department of Medicine, Stanford University School of Medicine, Stanford, California, USA

**Keywords:** drug repurposing, gene expression, transcriptomics, multi-cohort analysis, ulcerative colitis, electronic health records

## Abstract

**Objective:**

Ulcerative colitis (UC) is a chronic inflammatory disorder with limited effective therapeutic options for long-term treatment and disease maintenance. We hypothesized that a multi-cohort analysis of independent cohorts representing real-world heterogeneity of UC would identify a robust transcriptomic signature to improve identification of FDA-approved drugs that can be repurposed to treat patients with UC.

**Materials and Methods:**

We performed a multi-cohort analysis of 272 colon biopsy transcriptome samples across 11 publicly available datasets to identify a robust UC disease gene signature. We compared the gene signature to *in vitro* transcriptomic profiles induced by 781 FDA-approved drugs to identify potential drug targets. We used a retrospective cohort study design modeled after a target trial to evaluate the protective effect of predicted drugs on colectomy risk in patients with UC from the Stanford Research Repository (STARR) database and Optum Clinformatics DataMart.

**Results:**

Atorvastatin treatment had the highest inverse-correlation with the UC gene signature among non-oncolytic FDA-approved therapies. In both STARR (n = 827) and Optum (n = 7821), atorvastatin intake was significantly associated with a decreased risk of colectomy, a marker of treatment-refractory disease, compared to patients prescribed a comparator drug (STARR: HR = 0.47, *P* = .03; Optum: HR = 0.66, *P* = .03), irrespective of age and length of atorvastatin treatment.

**Discussion & Conclusion:**

These findings suggest that atorvastatin may serve as a novel therapeutic option for ameliorating disease in patients with UC. Importantly, we provide a systematic framework for integrating publicly available heterogeneous molecular data with clinical data at a large scale to repurpose existing FDA-approved drugs for a wide range of human diseases.

## INTRODUCTION

Ulcerative colitis (UC) is 1 of 2 main types of chronic idiopathic intestinal disorders that make up inflammatory bowel diseases (IBD). Nearly 1 million individuals are affected by UC in the US alone, with incidence and prevalence rising worldwide.^1–3^ UC is characterized by relapsing and remitting mucosal inflammation, starting from the rectum and extending to the entire colon.^1^ Therapeutic management of UC aims to induce and then maintain clinical and endoscopic remission. However, only about two-thirds of patients respond to corticosteroids,^4^ and a third of patients are nonresponders to anti-tumor necrosis factor (anti-TNF) treatment.^5^ Patients that are refractory to pharmacological treatment often require a colectomy to control their disease. The 10-year cumulative colectomy rate in patients with UC is estimated to be between 2.4%–10.4%.^6^^,^^7^ While total colectomy is the only known curative therapy, it is only performed as a last resort due to associated adverse side effects, including surgery-related complications such as clots, pouchitis, and bowel obstructions or strictures.^1^ The undesirability of colectomies underscores the need for additional medication options for patients with UC that can reduce colectomy rates.

Drug repositioning, or drug repurposing, is an effective strategy to find new indications for existing drugs. This strategy has been used with success across multiple diseases, including Parkinson’s disease,^8^ breast cancer,^9^ and colon cancer.^10^ Previously, a comparison of gene expression profiles from a compendium of 164 drug compounds with a gene expression signature of IBD derived from a single dataset of intestinal biopsies identified topiramate, an antiepileptic therapy, as a novel drug.^11^ However, despite *in vivo* data suggesting topiramate reduced gut inflammation,^11^ a subsequent analysis using insurance claims data was unable to find any association between topiramate use and various outcomes, including steroid use, biologic agent use, abdominal surgery, and hospitalization.^12^ These studies collectively emphasize the necessity of human data alongside a robust molecular signature of a disease.

Several studies have used transcriptomics to propose different molecular mechanisms that may contribute to UC pathology.^13–23^ However, these studies use only a single cohort, which typically does not capture the clinical and biological heterogeneity observed in the real-world patient population. This lack of biological and clinical heterogeneity in turn reduces the generalizability of findings. Using a multi-cohort analysis framework, we have repeatedly demonstrated that leveraging biologically, clinically, and technically heterogeneous cohorts identifies a more robust gene signature compared to using a single homogeneous cohort. This framework has been repeatedly used successfully to discover biomarkers that continue to validate in prospective studies.^24–27^ Here, we utilized this multi-cohort analysis approach to first identify a robust gene signature of UC, and then compared it against a set of transcriptome profiles of 781 FDA-approved small-molecule compounds. We identified atorvastatin as a potential drug in reverting the molecular signature of UC. Finally, we used 2 independent retrospective patient cohorts to demonstrate that atorvastatin exposure is correlated with decreased colectomy rates in patients with UC.

## MATERIALS AND METHODS

### Gene expression data collection and pre-processing

We searched the NCBI Gene Expression Omnibus (GEO)^28^ for gene expression datasets that profiled colon biopsies from patients with UC and non-IBD controls, defined as a normal colon negative for IBD and colorectal cancer, using the following terms: “(IBD OR colitis)” AND “Homo sapiens.” We identified and downloaded 11 gene expression datasets that contained 272 colon biopsy samples from patients with UC (N = 171) or healthy controls (N = 101) (Table 1).^13–23^

**Table 1.** Gene expression study cohort characteristics

Dataset	Accession Number	Disease state	Geographical location	Platform	Controls	Cases
Lepage et al.	GSE22619	Not reported	Lithuania; Germany	Affymetrix	10	10
Pekow et al.	GSE37283	Inactive	US	Affymetrix	5	15
Planell et al.	GSE38713	Inactive and active	Spain	Affymetrix	13	30
Ahrens et al.	GSE10191	Inactive and active	US	Affymetrix	11	8
Bjerrum et al.	GSE13367	Inactive and active	Denmark	Affymetrix	10	17
Mentero-Meléndez et al.	GSE36807	Inactive	Spain; US	Affymetrix	7	15
Galamb et al.	GSE4183	Active	Hungary	Affymetrix	8	9
Carey et al.	GSE9686	Inactive and active	US	Affymetrix	8	5
Kugathasan et al.	GSE10616	Not reported	US	Affymetrix	11	10
Arijs et al.	GSE16879	Active	Belgium	Affymetrix	6	24
Zhao et al.	GSE53306	Inactive and active	US	Illumina	12	28
**Total**	**11 datasets**		**8 countries**	**2 platforms**	**101**	**171**

### Integrative multi-cohort meta-analysis

We used the R package MetaIntegrator^29^ to apply 2 meta-analysis methods to combine (1) effect sizes and (2) *P* values as previously described.^24–27^ Briefly, we estimated the effect size of each gene within each dataset as Hedges’ adjusted *g* with correction for small sample size. For each gene, study-specific effect sizes were then combined into a summary effect size using a linear combination of study-specific effect sizes, *f_i_*, where each study-specific effect size was weighted by inverse of the variance in the corresponding study. After computing the summary effect size, *P* values were corrected for multiple hypotheses testing via Benjamini-Hochberg false discovery rate (FDR) correction.^30^ To avoid disproportionate influence of a single study and increase robustness, we selected the final UC gene signature by only including genes that remain statistically significant across all “leave-one-dataset-out” analyses and an FDR < 0.01. We then use the method described by Hedges and Pigott^31^ to compute statistical power for each gene and found that there was 99% statistical power for detecting differentially expressed genes with 1% type I error for summary effect size 0.66, 0.76, 0.95, and 1.41 in the presence of no, low, moderate, or high heterogeneity, respectively (Supplementary Figure 1). In total, we identified 2306 differentially expressed genes between patients with UC and healthy control samples.

### Pathway analysis

We performed overrepresentation pathway analysis^32^ using gene sets from Reactome database available through MSigDB.^33–35^ We eliminated pathways that contained fewer than 5 genes. We used Fisher’s exact test to calculate P values and determine significant pathways. We set the threshold for significant pathways with an FDR ≤ 5%.

### Computational prediction of novel UC therapies

For predicting FDA-approved drugs that can be repurposed to treat patients with IBD, we used lincsTools function in MetaIntegrator. We used transcriptome profiles from Library of Integrated Network-Based Cellular Signatures (LINCS)^36^ L1000 platform to compare drug signatures against our UC signature. We only used genes designated to be reproducible and self-connected (“gold”) by the Broad Institute in our analysis. We used Level 5 differential gene expression data from LINCS, which contains the effect sizes of all genes in a given cell line treated with a given perturbagen compared to controls. After filtering for FDA-approved small-molecule drugs, we performed Pearson correlations between each drug-UC signature pair. We corrected *P* values for multiple hypotheses using with Benjamini-Hochberg correction.^30^

### Analysis of patient records with UC

We used claims and electronic health record (EHR) databases for retrospective cohort analyses of patients with UC. All data was deidentified. The Stanford Research Repository (STARR) contains EHRs of 1.8 million adult and pediatric patients seen at Stanford University Medical Center from Jan 1, 2008, to Dec 31, 2015.^37^ Access was permitted through a previously approved IRB.^37^ The Optum Clinformatics DataMart is a national insurance claims database of 63 million US residents from Jan 1, 2004, to Dec 31, 2016^38^ (IRB-43693). Both databases capture fully adjudicated prescription, laboratory, medical, and hospital records.

### Cohort identification and assessment of exposures

The structure of the retrospective cohort study, with an active comparator design,^39–41^ from electronic health and claims records and subsequent statistical analyses were designed to emulate a target trial.^42^ Specifically, extra care must be taken with observational studies that involve statins, as long-term cardiovascular prescription drug users are often healthier^43^^,^^44^ than comparable patient cohorts, leading to deflated hazard estimates. Based on the recommendations for emulating a target trial,^42^ we selected patients with UC initiating atorvastatin therapy and a comparator group of IBD patients using other cardiovascular and lipomodulatory drugs. We excluded patients with a concurrent diagnosis of dysplasia, colorectal cancer, diverticulosis, or Crohn’s disease (ID9: 153.x, 230.3, 235.2, 239.0, 555.x, ICD 10: C18.x, K57.x, K51.x).

Next, we required that the patients with UC have a prescription for atorvastatin or a comparator therapy after the first recorded diagnosis of UC. The comparator drugs were prescription-only first or second line therapies intended for long-term treatment for cardiovascular conditions^45^ (benazepril, furosemide, losartan, propranolol, hydralazine) or lipid regulation^46^^,^^47^ (niacin, ezetimibe, cholestyramine, omega 3 fatty acids, metformin).

### Follow-up and outcome assessment

We censored patients at the last recorded date of insurance eligibility (Optum) or last recorded visit (STARR). The outcome for this study was a first-ever colectomy (Supplementary Table 1). We adjusted for potential confounding variables, including age, sex, and cardiovascular conditions and use of all comparator medications. We began our observation period at the first recorded prescription to mitigate the healthy user bias.^48^ We performed Cox proportional hazard modeling to estimate adjusted hazard ratios (HR) for the association between atorvastatin use in patients with UC and the primary outcome. All analyses were performed with the survival package (Version 1.1.4) in R (Version 3.6.1).

## RESULTS

### Multi-cohort analysis of colon biopsies from patients with UC identifies a robust gene signature

We chose to integrate multiple independent datasets that collectively represent biological, clinical, and technical heterogeneity observed in the real-world patient population to identify a robust gene signature for UC.^24–27^ Using NCBI GEO, we identified 11 whole transcriptome datasets containing 272 colon biopsies from patients with UC and healthy controls that met the inclusion criteria: at least 5 samples each of cases and controls, and samples must be from the colon (ileal samples were removed before analysis) (Table 1). Collectively, these datasets included patients from 8 countries (biological heterogeneity) with a wide range of disease severity (clinical heterogeneity) and profiled using different microarray platforms (technical heterogeneity).

**Figure 1.**
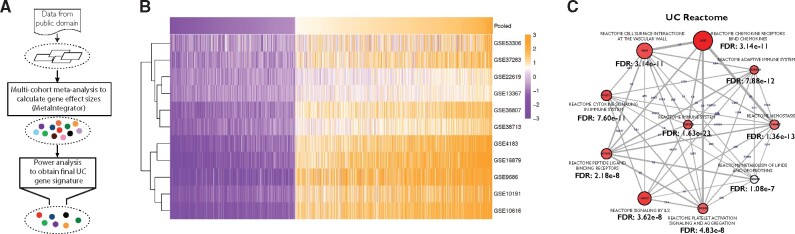
Multi-cohort meta-analysis identifies a robust UC gene signature. (A) Overview of multi-cohort analysis to identify UC gene signature. (B) Heatmap of the UC gene signature across all eleven datasets. (C) Top 10 statistically significant pathways using the Reactome pathway database. Number on an edge connecting 2 pathways represent the number of genes shared between the 2 pathways.

We used MetaIntegrator^29^^,^^49^ to analyze transcriptome profiles of 272 colon biopsy samples from healthy controls or patients with UC (Figure 1A). Using power analysis (see Materials and Methods and Supplementary Figure 1), we chose differentially expressed genes that met effect size thresholds appropriate for their between-study heterogeneity. We identified 2306 differentially expressed genes (1412 over-expressed, 894 under-expressed) (Figure 1B and Supplementary Table 2) including several genes that have been previously associated with UC. These genes include *THY1* (*CD90;* ES = 1.62, FDR = 1.49e–16) and *CDH1* (ES=−0.90, FDR = 8.32e–9) in genome-wide association studies; *S100A9* (ES = 1.80, FDR = 2.76e–8) and *S100A12* (ES = 1.12, FDR = 1.74e–10), which are both used as noninvasive markers of inflammation and diagnosing active IBD; metalloproteinases *MMP1* (ES = 2.02, FDR = 6.45e–18) and *MMP7* (ES = 1.71, FDR = 2.03e–9); and leukocyte-trafficking receptors *VCAM1* (ES = 1.19, FDR = 2.22e–7) and *ICAM1* (ES = 1.49, FDR = 9.64e–9) (Supplementary Figure 2). Importantly, we found between-study heterogeneity for these genes was low. Additionally, we did not find any clear trends based on the clusters identified by hierarchical clustering, including geographical location and disease activity. Consistent with previous findings, pathway analysis identified immune- and inflammation-related pathways including cytokine signaling, immunoregulatory interactions with adaptive immune system, and signaling by interleukins (ILs) (Figure 1C).^50^^,^^51^ These findings demonstrate our gene signature recapitulates known proinflammatory and immunomodulatory mechanisms underlying UC disease pathology.

### Identification of candidate drugs to treat UC by disease–drug associations

**Figure 2.**
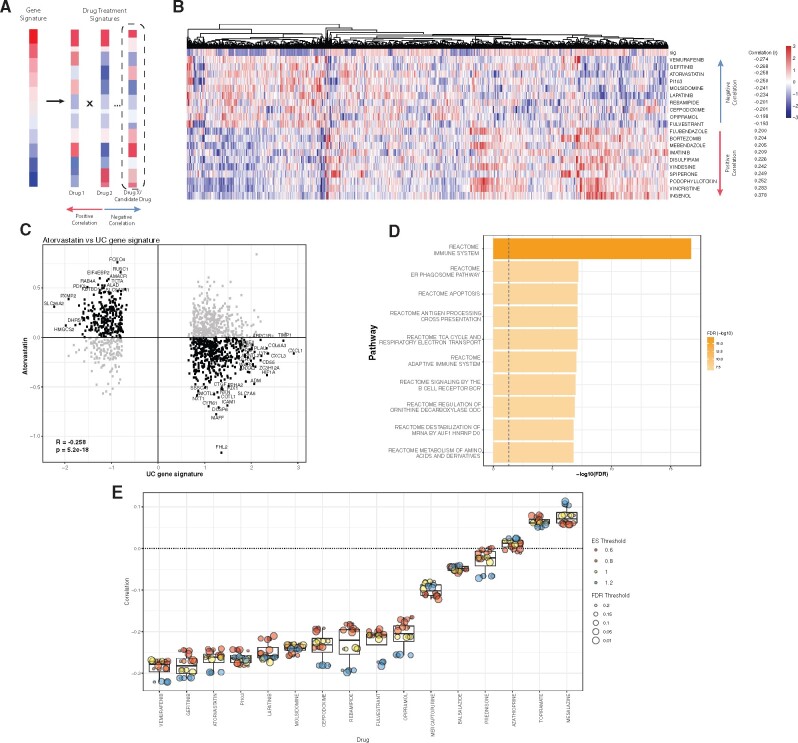
Disease–drug association analysis using LINCS perturbation database. (A) Schematic of the workflow for identifying candidate drugs that reverse UC signature. (B) Heatmap of the top 10 drug signatures inversely and positively correlated with disease signature. (C) Scatterplot of gene effect sizes in UC disease vs atorvastatin. (D) Pathway analysis using genes most significantly inverted between disease and drug signatures. FDR values are log10-scaled. (E) Sensitivity analysis of disease–drug correlations of FDA-approved small-molecule drugs. For each FDR and effect size threshold combination, a corresponding gene signature was generated. Pearson correlations were calculated between the disease signatures and each drug signature. Color represents log2-effect size threshold (0.6–1.2) and dot size represents FDR threshold (1%–20%).

We hypothesized that FDA-approved drugs with transcriptome profiles inversely correlated with our UC signature could reduce disease pathology. To test this hypothesis, we correlated the UC signature with transcriptome profiles of 781 FDA-approved small molecules from LINCS^36^ (see Materials and Methods; Figure 2A). One common biologic agent used to treat UC is infliximab, an anti-TNF^52^ monoclonal antibody. While LINCS did not generate *in vitro* transcriptome profiles using FDA-approved biologic agents, it did test various protein ligands to measure their effects on cell line gene expression, including TNF. Consistent with known association of TNF in IBD pathology, the TNF and UC gene signatures were positively correlated (mean r = 0.20, mean *P* = .001; Supplementary Figure 3). Additionally, both oncostatin M (OSM) and IL1 have been implicated in driving intestinal inflammation in patients with UC^53^ and were among the top 10 of all measured ligands.

Out of more than 19 000 small molecules profiled in the LINCS, we chose to consider only 781 FDA-approved molecules because these drugs have already proven to be largely safe for humans and are readily accessible for clinical use. Because we used only genes designated to be reproducible and self-connected (“gold”) by the Broad Institute (see **Materials and Methods**), not all genes were represented in the drug signatures; however, the shortlisted gene signature had similar classification capabilities as the original signature (Supplementary Figure 4). We calculated the Pearson correlation between the 1248 genes present in both the UC disease signature and each drug’s gene expression profile from LINCS (Figure 2B). The correlations ranged from −0.274 to 0.378. The 2 drugs with the highest inverse correlations, vemurafenib (r=−0.274; FDR: 2.6e–20) and gefitinib (r=−0.268, FDR = 2.0e–19), are both targeted cancer therapeutic agents. Vemurafenib is a targeted B-Raf enzyme inhibitor used in treating aggressive forms of melanoma.^54^ Gefitinib is an epidermal growth factor receptor (EGFR) inhibitor used in the treatment of cancers with overreactive or mutated EGFR.^55^ The drug with the third highest inverse correlation with the UC gene signature was atorvastatin (r=−0.258; FDR = 5.2e–18). Several studies have shown anti-inflammatory properties of statins in a variety of inflammatory contexts,^56–59^ which may explain the high significant inverse correlation between the atorvastatin and UC signatures. In line with these studies, further analysis of other statins revealed significant inverse drug correlations with our UC signature (Supplementary Table 2). We found that 731 genes (58%) out of 1248 were expressed in the opposite direction between UC and treatment with atorvastatin (Figure 2D;Supplementary Table 3). Pathway analysis of these genes found several overlapping pathways from the UC signature, including immune system and adaptive immune system (Figures 1C and 2D). Additionally, pathways such as apoptosis^60^^,^^61^ and regulation of ornithine decarboxylase ODC^62^ are known to play potential roles in IBD inflammation and pathology.

We performed sensitivity analysis to assess robustness of our results by changing the thresholds for FDR (1%–20%) and effect size (0.6–1.2) to select differentially expressed genes in UC (see **Materials and Methods**). Changing the stringency, irrespective of increasing or reducing, did not affect ranking of the top 10 FDA-approved drugs that were inversely correlated with the UC gene signature (Figure 2E). Several small-molecule compounds already approved by FDA for treating patients with UC were also inversely correlated with the UC gene signature, irrespective of which gene signature was used, though not all correlations were statistically significant. Topiramate is an FDA-approved antiepileptic drug previously identified as a potential therapeutic for patients with IBD.^11^ Interestingly, irrespective of the thresholds used for FDR or effect size, topiramate consistently showed very low positive correlation with our UC gene signature (Figure 2E).

### Atorvastatin use in patients with UC is associated with decreased risk of colectomy

Because vemurafenib and gefitinib are both targeted inhibitors for cancer, and are known to have serious adverse side effects,^63^ we decided to forego further investigation of these drugs and instead focused on atorvastatin. To examine the potential effects of atorvastatin on patients with UC, we estimated the risk of colectomy in atorvastatin users compared to patients who received a comparator drug (see Materials and Methods). We examined outcomes in patients with UC from Stanford University’s STARR EHR database and the Optum Clinformatics DataMart healthcare claims database.^38^ We structured our retrospective cohort analysis per recommended guidelines to best emulate a target trial (see Materials and Methods).^42^

The final study cohorts included 827 subjects in STARR (596 in the comparator group; 231 in the atorvastatin group), and 7821 subjects in Optum (4940 in the comparator group; 2881 in the atorvastatin group) (Table 2). The study characteristics of both cohorts are summarized in Table 2. The mean age was similar across both cohorts and groups, with those initiated on atorvastatin slightly older than comparator drug initiators in both STARR and Optum (STARR: 56.7 ± 16.5 years for comparator initiators, 62.5 ± 12.6 years for atorvastatin initiators; Optum: 55.5 ± 16.0 years for comparator initiators, 59.3 ± 12.8 years for atorvastatin initiators). UC-specific drug prescriptions^64^ were equivalent or higher in Optum compared to STARR.

**Table 2.** Demographic information on all cohorts of patients with UC

	STARR		Optum	
	Comparator	Atorvastatin	Comparator	Atorvastatin
	(n = 596)	(n = 231)	(n = 4940)	(n = 2881)
Age (mean (SD))	56.68 (16.15)	62.51 (12.61)	55.45 (16.04)	59.28 (12.76)
**Sex**				
Female	299 (50.2)	101 (43.7)	2593 (52.5)	1288 (44.7)
Male	297 (49.8)	130 (56.3)	2346 (47.5)	1591 (55.2)
Unknown	0 (0.0)	0 (0.0)	1 (0.0)	2 (0.1)
**Comparator Drug Rx**				
Niacin (%)	21 (3.5)	12 (5.2)	201 (4.1)	81 (2.8)
Ezetimibe (%)	9 (1.5)	8 (3.5)	436 (8.8)	177 (6.1)
Cholestyramine (%)	27 (4.5)	5 (2.2)	866 (17.5)	132 (4.6)
Omega FA (%)	44 (7.4)	22 (9.5)	241 (4.9)	71 (2.5)
Benazepril (%)	16 (2.7)	12 (5.2)	301 (6.1)	90 (3.1)
Furosemide (%)	181 (30.4)	50 (21.6)	1597 (32.3)	483 (16.8)
Losartan (%)	75 (12.6)	38 (16.5)	1055 (21.4)	385 (13.4)
Propranolol (%)	46 (7.7)	5 (2.2)	449 (9.1)	65 (2.3)
Metformin (%)	92 (15.4)	40 (17.3)	1186 (24.0)	505 (17.5)
Hydralazine (%)	119 (20.0)	33 (14.3)	150 (3.0)	72 (2.5)
**Comorbidities**				
CAD (%)	88 (14.8)	71 (30.7)	2023 (41.0)	1366 (47.4)
Cerebrovascular (%)	35 (5.9)	38 (16.5)	1695 (34.3)	1236 (42.9)
PVD (%)	53 (8.9)	23 (10.0)	952 (19.3)	680 (23.6)
CHF (%)	70 (11.7)	36 (15.6)	777 (15.7)	431 (15.0)
Colectomy (%)	68 (11.4)	10 (4.3)	141 (2.9)	42 (1.5)
Atorvastatin dose		29.64 (20.42)		27.11 (19.15)
(mean mg (SD))				
**IBD Rx**				
Mesalamine (%)	220 (36.9)	85 (36.8)	3303 (66.9)	1844 (64.0)
Olsalazine (%)	2 (0.3)	1 (0.4)	34 (0.7)	15 (0.5)
Balsalazide (%)	33 (5.5)	15 (6.5)	737 (14.9)	395 (13.7)
Sulfasalazine (%)	84 (14.1)	40 (17.3)	779 (15.8)	543 (18.8)
Mercaptopurine (%)	47 (7.9)	26 (11.3)	493 (10.0)	254 (8.8)
Azathioprine (%)	28 (4.7)	12 (5.2)	832 (16.8)	357 (12.4)
Infliximab (%)	28 (4.7)	11 (4.8)	395 (8.0)	173 (6.0)
Adalimumab (%)	8 (1.3)	5 (2.2)	254 (5.1)	119 (4.1)
Certolizumab (%)	0 (0.0)	0 (0.0)	26 (0.5)	18 (0.6)
Natalizumab (%)	0 (0.0)	0 (0.0)	2 (0.0)	1 (0.0)
Budesonide (%)	49 (8.2)	21 (9.1)	909 (18.4)	425 (14.8)
Prednisone (%)	245 (41.1)	92 (39.8)	3047 (61.7)	1608 (55.8)
Prednisolone (%)	147 (24.7)	55 (23.8)	1631 (33.0)	1017 (35.3)
Vedolizumab (%)	7 (1.2)	2 (0.9)	63 (1.3)	19 (0.7)
**Inflammatory Markers**				
Albumin (mean g/dL (SD))	3.55 (0.76)	3.70 (0.67)	4.55 (7.54)	4.98 (12.72)
CRP (mean mg/L (SD))	5.46 (9.08)	4.55 (5.84)	7.43 (6.03)	8.12 (6.74)

*Abbreviations:* CV, cardiovascular; CHF, chronic heart failure; CAD, coronary artery disease; PVD, peripheral vascular disease.

We calculated the Cox proportional hazard (see Materials and Methods) for first-ever colectomy in patients with UC treated with atorvastatin compared to those on a comparator therapy. There was a total of 78 colectomies in the STARR cohort (9.4%) and 183 colectomies in Optum (2.3%). The apparent discrepancy in colectomy rate can be explained by previously noted lower procedure reporting in claims data compared to EHRs.^65^ Epidemiological studies of UC patients with EHR data consistently report cumulative 10-year colectomy rates between 6.9 and 10.4%^6^^,^^66^^,^^67^ while a recent study using claims data reported a rate of 2.4%.^7^

**Figure 3.**
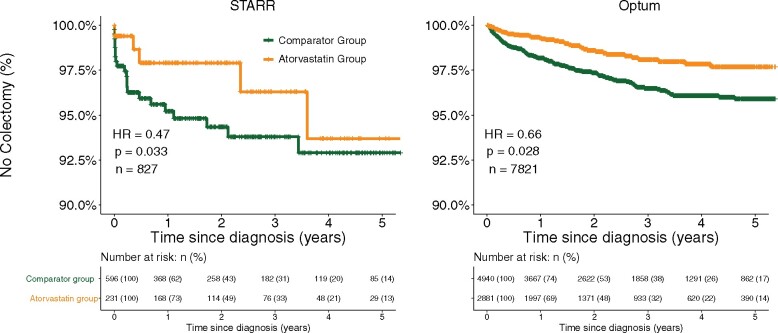
Kaplan-Meier curves of time to first colectomy of patients with UC who were on atorvastatin (yellow) or a comparator drug (green). (A) Stanford STARR cohort (n = 827). (B) Optum (n = 7821).

Patients with UC that were prescribed atorvastatin had significantly lower hazard ratios for colectomy rates in both the STARR and Optum cohorts (STARR HR: 0.47, *P* = .03; Optum HR: 0.66, *P* = .03) compared to those prescribed a comparator drug (Figure 3A and 3B). Atorvastatin continued to be associated with reduced rate of colectomy in patients with UC after adjusting for IBD therapies (STARR: HR = 0.43 [0.21–0.88], Optum: HR = 0.67 [0.46–0.97]). We also performed propensity-score matching, which revealed a significant overlap between the atorvastatin and comparator therapy without any matching in both STARR and Optum (Supplementary Figure 5). We used a default caliper of 0 on the logit scale, and the propensity scores were estimated by L1 regularized logistic regression using the MatchIt package (version 3.0.2; Supplementary Table 4). Atorvastatin use continued to confer protection from colectomy in both cohorts (Optum HR: 0.64 [0.41–0.88]; STARR HR: 0.46 [0.22–0.98]).

Atorvastatin use was associated with a longer time to first hospitalization after medication initiation compared to other cardiovascular and lipid-modulation therapies in Optum (Optum HR: 0.77, *P* < .001) but not in STARR (STARR HR: 0.97, *P* = .86; Table 3). Similarly, a small but statistically significant reduction in steroid use was seen in atorvastatin users in Optum (HR: 0.92, *P* = .003) but not in STARR (HR = 1.02, *P* = .89) (Table 3). No regression violated the proportional hazards assumption, as assessed by the cox.zph function from the R package survival. Further, we found that atorvastatin use did not confer protection for all outcomes. For example, atorvastatin use had no impact on pneumonia risk in either STARR or Optum (Supplementary Table 5**)**. We chose pneumonia because (1) there were sufficient cases to assess risk in STARR and Optum and (2) statin use has been shown to reduce the risk of pneumonia when the healthy-user bias is not considered.^68–70^ Thus, the lack of pneumonia protection due to statin use in our cohorts supports that the structure of this study minimizes the healthy user bias. To address the impact of time from diagnosis to drug initiation on outcomes, we included this time as a variable in our regression. This additional covariate did not increase the risk of colectomy in Optum (HR = 0.67 [0.46–0.97]). We next directly assessed the impact on colectomy risk by using time from diagnosis to therapy initiation as the sole variable in our cox regression. There was no relationship between this time and colectomy rate (HR = 0.99 [0.96–1.1]).

**Table 3.** Hazard ratios for adjusted and unadjusted primary and secondary outcomes

HR (95% CI)	*P* value	Adjusted for confounders
**STARR (n = 827)**		
*Colectomy*		
0.37 (0.19–0.73)	.004	No
0.47 (0.23–0.94)	.033	Yes
*First Hospitalization*		
0.85 (0.64–1.12)	.243	No
0.97 (0.72–1.32)	.863	Yes
*New Steroid Rx*		
0.95 (0.75–1.21)	.679	No
1.02 (0.78–1.33)	.889	Yes
**Optum (n = 7821)**		
*Colectomy*		
0.54 (0.38–0.77)	<.001	No
0.66 (0.45–0.95)	.028	Yes
*First Hospitalization*		
0.74 (0.68–0.80)	<.001	No
0.77 (0.71–0.84)	<.001	Yes
*New Steroid Rx*		
0.84 (0.80–0.89)	<.001	No
0.92 (0.87–0.97)	.002	Yes

### Evaluating the effects of other statins on colectomy rate

We also investigated whether other statins were associated with reduced colectomy rates in patients with UC. First, we compared transcriptome profiles of other statins available through LINCS and the UC signature. Each statin was negatively correlated with the UC signature, though less than atorvastatin, suggesting that other statins may have an effect similar to atorvastatin (Supplementary Table 2). In STARR, other statins were associated with reduced colectomy rates as well (HR: 0.41, 95% CI: 0.57–1.01; Supplementary Table 5) that was marginally significant (*P* = .067). Similarly, in Optum, colectomy rates were also lower for other statins (HR: 0.76, 95% CI: 0.57–1.01; Supplementary Table 5) that was marginally significant (*P* = .059). Although not statistically significant, these results suggest similar protective effect of other statins in the patients with UC.

### Sensitivity analysis of dose and duration of treatment

We did not require minimum time on therapy duration in either the atorvastatin or comparator drug cohorts to avoid the immortal time bias.^71^ This bias occurs when the definition of 1 group necessitates survival; for example, 6 months of statin treatment inherently requires the patient to survive at least that long. To examine the impact of medication dose and duration, we sequentially increased the minimum duration of treatment or atorvastatin dose in the patient cohort. There was no significant difference between any minimum dose or duration requirement (Supplementary Figure 6).

Additionally, we examined the outcomes of long-term compared to short-term atorvastatin use. We subset atorvastatin-treated patients to those that were followed for at least 720 days from the initiation of the drug (n = 1403/2881) and divided the patients into short-term (those prescribed atorvastatin for less than 6 months; n = 282) and long-term (those prescribed atorvastatin for more than 6 months; n = 1121). The median time on atorvastatin for the short-term cohort was 25.5 days and 1058 days (2.9 years) for the long-term cohort. Long-term use had lower rates of colectomy compared to short-term use (HR = 0.32 [0.11–0.95]; Supplementary Figure 7).

### Topiramate use does not convey protection from colectomy

Contrary to a previous study,^11^ our analysis of molecular data did not suggest topiramate as a potential therapeutic for patients with UC (Figure 2E). Additionally, topiramate was not significantly associated with colectomy rate in STARR (HR = 0.84, 95% CI = [0.26–2.77]) or Optum (HR = 1.00, 95% CI = [0.86–1.46]), in line with a separate retrospective cohort study.^12^

## DISCUSSION

There is a large body of literature devoted to molecular drug repurposing. Although FDA-approved, very few proposed therapies ever translate to clinical practice. In part, this is because the molecular signatures that are used to model the disease are based solely on a single cohort of patients and do not represent the biological and clinical heterogeneity observed in the real-world patient population. Additionally, few studies leverage existing patient data to preliminarily examine any putative treatments. We sought to address these shortcomings in 2 ways.

First, we utilized our multi-cohort analysis framework that has been repeatedly demonstrated to produce robust gene signatures across diseases by leveraging biological, clinical, and technical heterogeneity across multiple independent datasets. By integrating 11 gene expression datasets, we increased the probability that our gene signature is representative of the common disease state across a heterogeneous group of patients with UC.^25–27^^,^^29^^,^^72^

Second, we validated our putative drug in 2 independent retrospective patient cohorts using recently published guidelines to best emulate a target trial in retrospective studies.^42^ Long-term cardiovascular prescription drug users are often healthier^43^^,^^44^ and have better healthcare utilization metrics^73^ than comparable patient cohorts. Therefore, we compared atorvastatin users to patients with UC treated with cardiovascular or lipid-modulating drugs intended for long-term therapy. We began our observation period at the first recorded prescription to mitigate the healthy user bias.^48^ When designing a retrospective study, extra care must be taken to not include information about therapy duration into the initial inclusion criteria, as this information would not be available in a prospective study.^74^ Inclusion of this information can produce the immortal time bias, which has been shown to artificially deflate hazard ratios.^71^^,^^74^^,^^75^ Therefore, we did not require a certain duration of drug treatment to be included in this study. A previous study from Dudley et al^11^ used a single cohort and found a significant inverse correlation between their disease signature and topiramate, an anticonvulsant drug, while our analysis found a nonsignificant positive correlation with our disease signature. Although reversing a disease’s expression signature may not be representative of all mechanisms by which putative drugs may prove efficacious in a disease, the inverse correlation of atorvastatin treatment from the molecular data matched the protective effects seen in the clinical data, highlighting the importance of using patient data when validating putative drug targets. Together, the molecular and clinical data in this study strongly suggest that long-term atorvastatin use is associated with reduced rate of colectomy in patients with UC.

Atorvastatin is commonly prescribed for its lipid-lowering effect through inhibition of 3-hydroxy-3-methylglutaryl coenzyme A reductase (HMG-CoA reductase). Additionally, atorvastatin along with other statins, has been shown to have anti-inflammatory and proapoptotic effects. These include downregulation of molecular mediators involved in IBD-specific inflammation and reduction of colitis in animal models of IBD.^57–59^ Direct clinical studies of atorvastatin on UC remain sparse and conflicting. Two small studies have offered differing results on the protective effect of atorvastatin on patients with UC.^76^^,^^77^ Dhamija et al^77^ examined the potential for atorvastatin to treat acute exacerbation of UC. They followed patients for 8 weeks and found no evidence for protection against acute exacerbation. However, Higgins et al^76^ concluded that atorvastatin treatment conferred a positive effect on UC disease outcome after a 24-week follow-up period. Two small uncontrolled trials of atorvastatin treatment in Crohn’s Disease (CD) patients demonstrated a measurable reduction in proinflammatory markers and a statistically nonsignificant decrease in disease activity on treatment.^58^^,^^60^ A retrospective study concluded that statin use was associated with a reduction of oral steroid use.^78^ Another study suggested that statin exposure was associated with decreased risk of new onset IBD.^79^ We hypothesize that although atorvastatin may not work as a short-term alternative to steroid to combat acute inflammatory flares, it may have potential long-term benefits. Indeed, we show that long-term atorvastatin use conferred increased protection compared to short-term atorvastatin use (Supplementary Figure 7), and this effect has been noted in atorvastatin suppression of other autoinflammatory diseases.^24^^,^^78^

While the exact mechanism of action of the anti-inflammatory properties of atorvastatin are not well-defined, many groups have provided *in vitro and in vivo* evidence pointing to a variety of pathways in which atorvastatin may derive its pleiotropic properties, including TNF, CXCL10, and MCP-1/CCL2.^56^^,^^58^ In our analysis, some of the most inversely expressed genes between UC and atorvastatin included *CXCL1*, *CXCL3*, and *ICAM1* (Figure 2C). Previous studies have shown increased expression of *CXCL1 and CXCL3* in both a rodent model of UC as well as inflamed samples from patients with UC.^79^^,^^80^ Preclinical trials blocking ICAM1 using antibodies have shown therapeutic benefit in models of colitis, and no human clinical trials have been conducted using antibodies against ICAM1.^81^ While our study was primarily focused on atorvastatin, other medications or ligands such as rebamipide and oncostatin M were included in our findings that have known roles in mucosal healing^82^ or inflammation.^53^ Overall, our study suggests potential novel pathways by which atorvastatin could be acting. Further studies are required to confirm whether our *in-silico* findings are generalizable to *in vivo* models.

Although the EHR data corroborate the results we obtained from gene expression analysis, there are some caveats to our study. While the LINCS database contains data for many perturbagens, all of the cells tested were derived from immortalized cell lines, which could bias the scope of drug effects seen in the gene expression data. While most known therapies currently in use to treat UC demonstrated an inverse correlation with our disease signature, mesalazine showed a statistically insignificant positive correlation. Mesalazine is enzymatically processed by N-acetyltransferase, primarily in the liver and intestinal mucosa, into its active metabolite, N-acetyl-5- aminosalicylic acid.^83^ It is this active metabolite that conveys nearly all of mesalazine’s anti-inflammatory properties, which may not be actively present in cell lines. Additionally, given the limitations of administrative data, we were unable to examine the potentially important effects of disease phenotype, smoking history, or use of nonprescription drugs in our cohort studies. We recognize that colectomy rate may not be the perfect marker of disease severity in patients with UC; however, colectomies are an important clinical outcome in and of itself given the economic burden and undesired side effects of this procedure.

In summary, we demonstrate that a robust statistical approach leveraging multiple public gene expression microarray datasets can be used to infer novel drug therapies for patients with UC and offer nationwide EHR and claims data to support the association of atorvastatin with the amelioration of disease. Because atorvastatin is already recognized as a safe and effective drug for treating cardiovascular disease in humans, and has milder side effect profile compared to many other current UC drugs,^84^ our results support additional investigation into the use of atorvastatin for treating patients with UC. Prospective controlled clinical trials are needed to confirm whether atorvastatin treatment would benefit patients with UC. Finally, we describe a framework for integrating large-scale heterogeneous molecular and clinical data that can be used for other diseases, especially ones with no FDA-approved drug available for treatment.

## FUNDING

LB is funded by the Stanford Bio-X Graduate Fellowship. MKDS is funded in part by the National Heart, Lung, and Blood Institute (F30 n) and The Stanford University Medical Scientist Training Program (T32GM007365). PK is funded in part by the Bill and Melinda Gates Foundation (OPP1113682); the National Institute of Allergy and Infectious Diseases (NIAID) grants 1U19AI109662, U19AI057229, and 5R01AI125197; Department of Defense contracts W81XWH-18-1-0253 and W81XWH1910235; and the Ralph & Marian Falk Medical Research Trust, outside of this work.

## AUTHOR CONTRIBUTIONS

PK, LB, and MKDS conceived the study, interpreted the data, and wrote the manuscript. LB and MKDS acquired, processed, and analyzed molecular and clinical data, respectively. ES assisted with the processing of clinical data. LK assisted with some of the molecular analyses. AH advised regarding clinical relevance and edited the manuscript. NHS advised regarding cohort design, analysis of clinical data, and edited the manuscript. PK supervised the study.

## DATA AVAILABILITY STATEMENT

All disease gene expression datasets used in this study were derived from the public domain via NCBI GEO (https://www.ncbi.nlm.nih.gov/geo/). Individual dataset URLs can be found in Supplementary Table 1. Drug transcriptomic data were obtained through the publicly available LINCS database (https://clue.io/lincs); aggregated LINCS data used in this study are available through R using the *MetaIntegrator* package (version 2.1.3) lincsTools function. The clinical data used (both Stanford STARR and Optum) cannot be shared due to potential ethical/privacy concerns.

## SUPPLEMENTARY MATERIAL

Supplementary material is available at *Journal of the American Medical Informatics Association* online.

## CONFLICT OF INTEREST STATEMENT

None declared.

## Supplementary Material

ocab165_Supplementary_Data
