# Supplementary material for: Computational drug repositioning of atorvastatin for ulcerative colitis
Source: J Am Med Inform Assoc. 2021 Sep 16;28(11):2325–35. doi: 10.1093/jamia/ocab165 (PMC8510297; doi:10.1093/jamia/ocab165)
Supplement: ocab165_Supplementary_Data [file ocab165_supplementary_data.zip › Supplemental_Figures.docx]

**Supplementary Figure 2.** Forest plots of genes previously associated with UC are highly conserved across datasets. The X axes represent standardized mean difference between UC and healthy control samples, computed as Hedges’ *g,* in log2 scale. The size of the blue rectangles is inversely proportional to the SEM in the study. Whiskers represent the 95% confidence interval. The red diamonds represent the combined mean difference for a given gene across all datasets. Width of a diamond represents the 95% confidence interval of overall mean difference.

**Supplementary Figure 1.** Power analysis calculation incorporating heterogeneity results in different effect size cutoffs for varying levels of heterogeneity.

**Supplementary Figure 4**. ROC plots of the UC gene signature. (A) ROC of the full gene signature with the ES threshold determined by power analysis. (B) The final signature, representing the 1248 gene subset that is present in the LINCs platform out of the original signature of 2306 genes.

**Supplementary Figure 3**. Sensitivity analysis of disease-drug correlations of highest correlated ligands. For each FDR and effect size threshold combination, a corresponding gene signature was generated. Pearson correlations were calculated between the disease signatures and each ligand signature. Color represents log2-effect size threshold (0.6-1.2) and dot size represents FDR threshold (1%-20%).

**Supplementary Figure 5**. Propensity-score similarity metrics. A) shows matching metrics before and after 1-to-1 propensity matching for STARR. B) shows matching metrics before and after performing 1-to-1 propensity matching for Optum.


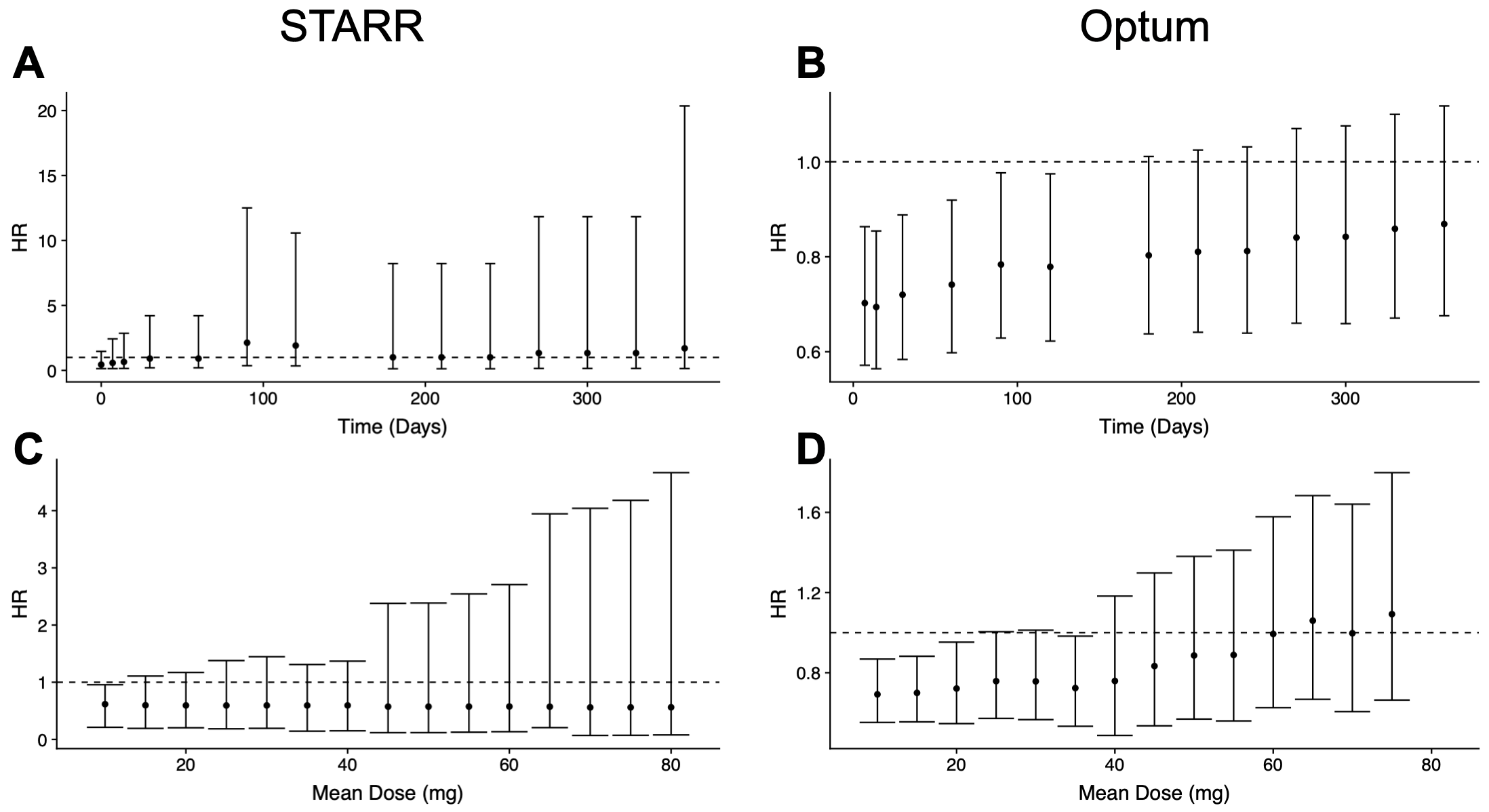
**Supplementary Figure 6**. HR sensitivity to duration of statin treatment in STARR (A) and Optum (B). Drug dose sensitivity analysis in STARR (C) and Optum (D).

**Supplementary Figure 7**. Kaplan-Meier curve between subjects taking atorvastatin long-term versus short-term in the Optum cohort. Long-term use is defined as > 60 days of prescription for the subject.
