# Supplementary material for: Computational drug repositioning of atorvastatin for ulcerative colitis
Source: J Am Med Inform Assoc. 2021 Sep 16;28(11):2325–35. doi: 10.1093/jamia/ocab165 (PMC8510297; doi:10.1093/jamia/ocab165)
Supplement: ocab165_Supplementary_Data [file ocab165_supplementary_data.zip › SuppTable4.pdf]

	Control	Treated
All	596	231
Matched	231	231
Unmatched	365	0
Discarded	0	0

Unmatched

	Means Treat	Means Contr	SD Control	Mean Diff	eQQ Med	eQQ Mean	eQQ Max
distance	0.364	0.247	0.141	0.117	0.110	0.117	0.180
Age	62.510	56.676	16.153	5.834	5.293	5.903	15.827
Sex: Female	0.437	0.502	0.500	-0.064	0.000	0.065	1.000
Sex: Male	0.563	0.498	0.500	0.064	0.000	0.065	1.000
mesalamine	0.368	0.369	0.483	-0.001	0.000	0.000	0.000
olsalazine	0.004	0.003	0.058	0.001	0.000	0.000	0.000
balsalazide	0.065	0.055	0.229	0.010	0.000	0.009	1.000
sulfasalazine	0.173	0.141	0.348	0.032	0.000	0.030	1.000
mercaptopur	0.113	0.079	0.270	0.034	0.000	0.035	1.000
azathioprine	0.052	0.047	0.212	0.005	0.000	0.004	1.000
infliximab	0.048	0.047	0.212	0.001	0.000	0.000	0.000
adalimumab	0.022	0.013	0.115	0.008	0.000	0.009	1.000
budesonide	0.091	0.082	0.275	0.009	0.000	0.009	1.000
prednisone	0.398	0.411	0.492	-0.013	0.000	0.013	1.000
prednisolone	0.238	0.247	0.431	-0.009	0.000	0.009	1.000
niacin	0.052	0.035	0.185	0.017	0.000	0.017	1.000
ezetimibe	0.035	0.015	0.122	0.020	0.000	0.017	1.000
cholestyrami	0.022	0.045	0.208	-0.024	0.000	0.026	1.000
omega	0.095	0.074	0.262	0.021	0.000	0.022	1.000
benazepril	0.052	0.027	0.162	0.025	0.000	0.026	1.000
furosemide	0.216	0.304	0.460	-0.087	0.000	0.087	1.000
losartan	0.165	0.126	0.332	0.039	0.000	0.039	1.000
metformin	0.173	0.154	0.362	0.019	0.000	0.017	1.000
hydralazine	0.143	0.200	0.400	-0.057	0.000	0.056	1.000
propranolol	0.022	0.077	0.267	-0.056	0.000	0.056	1.000
coronary_art	0.307	0.148	0.355	0.160	0.000	0.160	1.000
cerebrovascu	0.165	0.059	0.235	0.106	0.000	0.104	1.000
periph_vascu	0.100	0.089	0.285	0.011	0.000	0.009	1.000
CHF	0.156	0.117	0.322	0.038	0.000	0.039	1.000

Matched

	Means Treat	Means Contr	SD Control	Mean Diff	eQQ Med	eQQ Mean	eQQ Max
distance	0.364	0.352	0.149	0.011	0.001	0.012	0.066
Age	62.510	61.708	14.523	0.802	1.570	1.976	8.493
Sex: Female	0.437	0.468	0.500	-0.030	0.000	0.030	1.000
Sex: Male	0.563	0.532	0.500	0.030	0.000	0.030	1.000
mesalamine	0.368	0.411	0.493	-0.043	0.000	0.043	1.000
olsalazine	0.004	0.009	0.093	-0.004	0.000	0.004	1.000
balsalazide	0.065	0.069	0.254	-0.004	0.000	0.004	1.000
sulfasalazine	0.173	0.173	0.379	0.000	0.000	0.000	0.000
mercaptopur	0.113	0.117	0.322	-0.004	0.000	0.004	1.000
azathioprine	0.052	0.039	0.194	0.013	0.000	0.013	1.000
infliximab	0.048	0.069	0.254	-0.022	0.000	0.022	1.000
adalimumab	0.022	0.026	0.159	-0.004	0.000	0.004	1.000
budesonide	0.091	0.113	0.317	-0.022	0.000	0.022	1.000
prednisone	0.398	0.420	0.495	-0.022	0.000	0.022	1.000
prednisolone	0.238	0.264	0.442	-0.026	0.000	0.026	1.000
niacin	0.052	0.056	0.231	-0.004	0.000	0.004	1.000
ezetimibe	0.035	0.030	0.172	0.004	0.000	0.004	1.000
cholestyrami	0.022	0.022	0.146	0.000	0.000	0.000	0.000
omega	0.095	0.108	0.311	-0.013	0.000	0.013	1.000
benazepril	0.052	0.043	0.204	0.009	0.000	0.009	1.000
furosemide	0.216	0.203	0.403	0.013	0.000	0.013	1.000
losartan	0.165	0.152	0.359	0.013	0.000	0.013	1.000
metformin	0.173	0.195	0.397	-0.022	0.000	0.022	1.000
hydralazine	0.143	0.139	0.346	0.004	0.000	0.004	1.000
propranolol	0.022	0.013	0.113	0.009	0.000	0.009	1.000
coronary_art	0.307	0.299	0.459	0.009	0.000	0.009	1.000
cerebrovascu	0.165	0.130	0.337	0.035	0.000	0.035	1.000
periph_vascu	0.100	0.091	0.288	0.009	0.000	0.009	1.000
CHF	0.156	0.147	0.355	0.009	0.000	0.009	1.000

Percent Balance Improvement

	Mean Diff.	eQQ Med	eQQ Mean	eQQ Max
distance	90.210	99.391	89.868	63.103
Age	0.236	0.193	0.182	0.127
Sex: Female	52.981	0.000	53.333	0.000
Sex: Male	52.981	0.000	53.333	0.000
mesalamine	-3625.000	0.000	-Inf	-Inf
olsalazine	-344.776	0.000	-Inf	-Inf
balsalazide	54.746	0.000	50.000	0.000
sulfasalazine	100.000	0.000	100.000	100.000
mercaptopur	87.152	0.000	87.500	0.000
azathioprine	-161.404	0.000	-200.000	0.000
infliximab	-3286.364	0.000	-Inf	-Inf
adalimumab	47.350	0.000	50.000	0.000
budesonide	-148.956	0.000	-150.000	0.000
prednisone	-69.030	0.000	-66.667	0.000
prednisolone	-203.823	0.000	-200.000	0.000
niacin	74.098	0.000	75.000	0.000
ezetimibe	77.836	0.000	75.000	0.000
cholestyrami	100.000	0.000	100.000	100.000
omega	39.349	0.000	40.000	0.000
benazepril	65.509	0.000	66.667	0.000
furosemide	85.114	0.000	85.000	0.000
losartan	66.410	0.000	66.667	0.000
metformin	-15.147	0.000	-25.000	0.000
hydralazine	92.379	0.000	92.308	0.000
propranolol	84.410	0.000	84.615	0.000
coronary_art	94.579	0.000	94.595	0.000
cerebrovascu	67.259	0.000	66.667	0.000
periph_vascu	18.635	0.000	0.000	0.000
CHF	77.450	0.000	77.778	0.000
