# Supplementary material for: Computational drug repositioning of atorvastatin for ulcerative colitis
Source: J Am Med Inform Assoc. 2021 Sep 16;28(11):2325–35. doi: 10.1093/jamia/ocab165 (PMC8510297; doi:10.1093/jamia/ocab165)
Supplement: ocab165_Supplementary_Data [file ocab165_supplementary_data.zip › SuppTable5.docx]

|  | **STARR** | **Optum** |
| --- | --- | --- |
|  | (n = 247) | (n = 3174) |
| Pravastatin | 61 (24.7%) | 772 (24.3%) |
| Simvastatin | 119 (48.1%) | 1607 (50.6%) |
| Rosuvastatin | 47 (19.0%) | 538 (16.9%) |
| Lovastatin | 20 (8.1%) | 257 (8.1%) |

| **HR (95% CI)** | **p-value** | **Adjusted for Confounders** |  |
| --- | --- | --- | --- |
|  |  |  |  |
| **Atorvastatin Use - Pneumonia** |  |  |  |
| *STARR (n = 827)* |  |  |  |
| *Atorvastatin users (n = 231)* |  |  |  |
| *Comparator drug users (n = 596)* |  |  |  |
| 0.87 (0.48-1.59) | 0.65 | No |  |
| 0.74 (0.43-1.34) | 0.54 | Yes |  |
| *Optum (n = 7821)* |  |  |  |
| *Other statin users (n = 2881)* |  |  |  |
| *Comparator drug users (n = 4940)* |  |  |  |
| 0.90 (0.62-1.30) | 0.57 | No |  |
| 1.12 (0.76-1.66) | 0.55 | Yes |  |
| **All Statin Use - Colectomy** |  |  |  |
| *STARR (n = 843)* |  |  |  |
| *Other statin users (n = 247)* |  |  |  |
| *Comparator drug users (n = 596)* |  |  |  |
| 0.34 (0.13-0.88) | 0.026 | No |  |
| 0.41 (0.57-1.01) | 0.067 | Yes |  |
| *Optum (n = 8114)* |  |  |  |
| *Other statin users (n = 3174)* |  |  |  |
| *Comparator drug users (n = 4940)* |  |  |  |
| 0.60 (0.46-0.78) | <0.001 | No |  |
| 0.76 (0.57-1.01) | 0.059 | Yes |  |
